# P-1707. A Comparative Analysis of Clindamycin versus Linezolid as Adjunctive Anti-toxin Therapy for Invasive Group A *Streptococcal* Infections

**DOI:** 10.1093/ofid/ofae631.1873

**Published:** 2025-01-29

**Authors:** Megha Jagannathan, Tamara Jordan, Daniel Kinsey, Rachel M Kenney, Michael Veve, Anita Shallal, Geehan Suleyman

**Affiliations:** Henry Ford Hospital, Detroit, Michigan; Henry Ford Hospital, Detroit, Michigan; Henry Ford Hospital, Detroit, Michigan; Henry Ford Hospital, Detroit, Michigan; Henry Ford Health, Detroit, Michigan; Henry Ford Health, Detroit, Michigan; Henry Ford Health, Detroit, Michigan

## Abstract

**Background:**

Group A *Streptococcus* (GAS) is an important pathogen that can cause life-threatening disease. Clindamycin (DA) and linezolid (LZD) have been used as adjunctive antitoxin (AT) therapy in high-inoculum GAS infections to inhibit bacterial protein synthesis. However, there is concern about DA efficacy in the era of increasing DA resistance, where LZD may have a role. We evaluated outcomes of patients with invasive GAS infection who received DA or LZD.

**Table 1.**
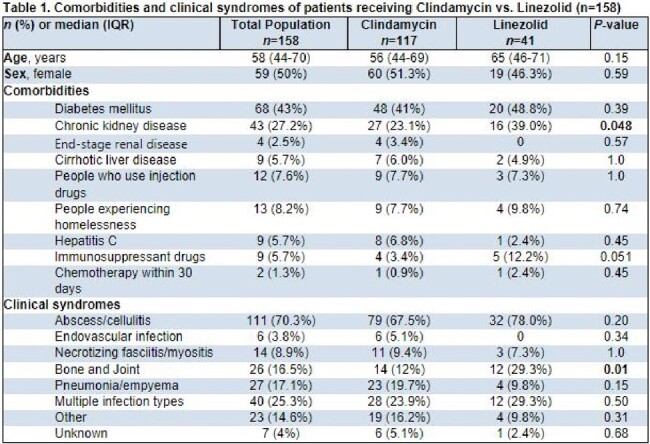
Comorbidities and clinical syndromes of patients receiving Clindamycin vs. Linezolid (n=158)

**Methods:**

Retrospective cohort study comparing patients with positive blood cultures (BC) for GAS from June 2013-Dec 2023 treated with DA or LZD ≥ 48 hours. We identified patients using a data query for positive BC for GAS through Microsoft SQL. Patients aged < 18 years, or those with polymicrobial bacteremia, receipt of both AT therapies, incomplete data, or enrolled in hospice/died within 48-hours of admission were excluded. Collected variables included: demographics, infection characteristics, microbiologic data, adjunct therapy (surgical, immunoglobulin), and clinical outcomes (treatment-associated adverse events, 30-day all-cause mortality and infection-related readmission).

**Table 2.**
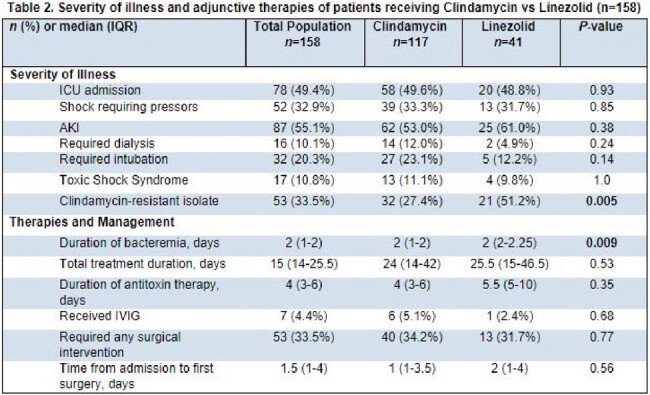
Severity of illness and adjunctive therapies of patients receiving Clindamycin vs Linezolid (n=158)

**Results:**

158 patients were included; 117 patients received DA and 41 patients LZD. Baseline characteristics were similar among groups except for chronic kidney disease, which was more common in the LZD group (Table 1). The most common clinical syndrome accompanying bacteremia in both groups was abscess/cellulitis; bone and joint infection was more prevalent in the LZD group. 55 (33.5%) of GAS isolates were DA resistant. There was no significant difference in severity of illness, surgical interventions, or duration of therapy between the two groups (Table 2). Duration of bacteremia was significantly longer in the LZD group. There was no significant difference in readmission (10.3% vs 12.2%, p=0.77) or all-cause mortality within 30 days (17.1% vs 7.3%, p=0.13) in the DA versus LZD groups. Treatment-associated adverse events were low across both groups [Figure 1].

**Figure:**
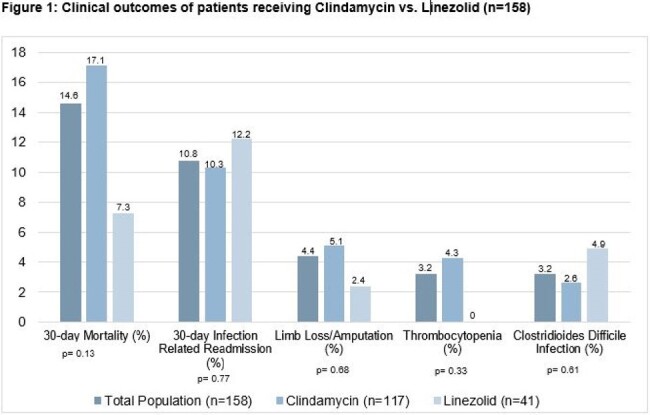

**Conclusion:**

Despite increasing DA resistance at our facility, there was no significant difference in outcomes between patients treated with LZD vs DA plus standard therapy, consistent with prior literature. Further studies are needed to determine optimal therapy for invasive GAS.

**Disclosures:**

**Rachel M. Kenney, PharmD, BCIDP**, Medtronic Inc: Spouse is an employee, stockholder

